# Genome wide SNP discovery in flax through next generation sequencing of reduced representation libraries

**DOI:** 10.1186/1471-2164-13-684

**Published:** 2012-12-06

**Authors:** Santosh Kumar, Frank M You, Sylvie Cloutier

**Affiliations:** 1 Cereal Research Centre, Agriculture and Agri-Food Canada, 195 Dafoe Road, Winnipeg, Manitoba, R3T 2M9, Canada; 2 Department of Plant Science, University of Manitoba, 66 Dafoe Road, Winnipeg, Manitoba, R3T 2N2, Canada

**Keywords:** Single nucleotide polymorphism (SNP), Genotyping-by-sequencing (GBS), Reduced representation library (RRL), Illumina, Flax, *Linum usitatissimum*, AGSNP

## Abstract

**Background:**

Flax (*Linum usitatissimum* L.) is a significant fibre and oilseed crop. Current flax molecular markers, including isozymes, RAPDs, AFLPs and SSRs are of limited use in the construction of high density linkage maps and for association mapping applications due to factors such as low reproducibility, intense labour requirements and/or limited numbers. We report here on the use of a reduced representation library strategy combined with next generation Illumina sequencing for rapid and large scale discovery of SNPs in eight flax genotypes. SNP discovery was performed through *in silico* analysis of the sequencing data against the whole genome shotgun sequence assembly of flax genotype CDC Bethune. Genotyping-by-sequencing of an F_6_-derived recombinant inbred line population provided validation of the SNPs.

**Results:**

Reduced representation libraries of eight flax genotypes were sequenced on the Illumina sequencing platform resulting in sequence coverage ranging from 4.33 to 15.64X (genome equivalents). Depending on the relatedness of the genotypes and the number and length of the reads, between 78% and 93% of the reads mapped onto the CDC Bethune whole genome shotgun sequence assembly. A total of 55,465 SNPs were discovered with the largest number of SNPs belonging to the genotypes with the highest mapping coverage percentage. Approximately 84% of the SNPs discovered were identified in a single genotype, 13% were shared between any two genotypes and the remaining 3% in three or more. Nearly a quarter of the SNPs were found in genic regions. A total of 4,706 out of 4,863 SNPs discovered in Macbeth were validated using genotyping-by-sequencing of 96 F_6_ individuals from a recombinant inbred line population derived from a cross between CDC Bethune and Macbeth, corresponding to a validation rate of 96.8%.

**Conclusions:**

Next generation sequencing of reduced representation libraries was successfully implemented for genome-wide SNP discovery from flax. The genotyping-by-sequencing approach proved to be efficient for validation. The SNP resources generated in this work will assist in generating high density maps of flax and facilitate QTL discovery, marker-assisted selection, phylogenetic analyses, association mapping and anchoring of the whole genome shotgun sequence.

## Background

Flax (*Linum usitatissimum* L.) is a self-pollinated annual species (2n = 2x = 30) belonging to the Linaceae family. It has been utilised by mankind for some 30,000 years (Paleolithic era) [1], was domesticated ~7,000 years ago in the Near East and then spread to the Fertile Crescent where it was grown for its seed oil and stem fibres [2]. Currently, Canada is the world’s largest producer of linseed (http://publications.gc.ca/collections/collection_2011/statcan/22-007-X/22-007-2011002-eng.pdf).

Flax oil is highly sought after in the fabrication of biodegradable products such as paint, linoleum and varnish, while its oil-free meal is used as livestock feed. Recently, linseed has gained importance as nutraceutical primarily because of its α-linolenic acid (ALA) and lignan content. The ALA component of flax oil (omega-3 fatty acid) improves bone and cardio-vascular health [3-5] while lignans are a rich source of antioxidants and precursors of various hormones [6]. Animal feed for cattle and chicken is being fortified with flax to produce omega-3 enriched meat and eggs [7].

To assess and capitalize upon the genetic variability in flax, genomic resources are needed. The flax genome assembled from short shotgun reads [8] as well as a collection of expressed sequence tags (ESTs) from more than 10 different tissue libraries are now available [9]. Genetic mapping remains a commonly used approach to understand the molecular basis of phenotypic traits. Various molecular markers including random amplified polymorphic DNA (RAPD), restriction fragment length polymorphism (RFLP), amplified fragment length polymorphism (AFLP) and simple sequence repeat (SSR) have been developed to analyse flax genetic diversity [10-19]. Three bi-parental population-based linkage maps of flax have been published to date: an AFLP map of 213 markers [10], an RFLP and RAPD map of 94 markers [12] and an SSR map of 113 markers [18]. A recently constructed 770 SSR consensus map based on three populations constitutes a significant improvement over previous maps but even this marker density remains insufficient for many applications [19]. An ideal molecular approach to generate markers is one that assesses numerous reliable markers covering the entire genome in a single and simple experiment [20]. The discovery of single nucleotide polymorphic (SNP) markers combined with next generation sequencing (NGS) permits the identification of thousands of markers from entire genomes which can be used for linkage map construction, genetic diversity analyses, marker-trait association and marker-assisted selection [21]. SNPs have been discovered by high throughput sequencing in humans [22], *Drosophila melanogaster*[23], wheat [24], eggplant [25], rice [26-28], *Arabidopsis thaliana*[29,30], barley [31-33], walnut [34], lupin [35], globe artichoke [36], rapeseed [37], perennial ryegrass [38] and maize [39] to name but a few. SNP discovery through genome sequencing is readily accomplished in simpler genomes like rice and *Arabidopsis*[28,40] but the task remains challenging for a number of economically important crops [41,42]. The discovery process is also impeded by the presence of repeat elements, paralogous sequences and reference genomes that are incomplete or inaccurate. The flax genome of CDC Bethune has an estimated size of ~370 Mbp with a high proportion of low copy sequences [43]. Its repetitive fraction consists of ribosomal DNA (~13.8%), known transposable elements (~6.1%) and putative novel repeat elements (~7.4%) [44] making it highly suitable for SNP discovery.

Genomic complexity can be reduced using restriction enzymes [22], high-Cot selection [45], methylation filtration [46], microarrays [47,48] and cDNAs [49]. Trebbi et al. have described the pros and cons of these methods [50]. The use of reduced representation libraries (RRL) is advantageous because the reduction of genome complexity can be altered by selecting different enzymes or size ranges. RRL sequencing, first proposed for the human genome, reduces genome complexity, facilitates re-sampling and generates sufficient coverage for accurate SNP calling [22]. Deep re-sequencing of RRLs using the sequencing-by-synthesis method has been performed for the purpose of SNP discovery in soybean and sorghum [51,52].

SNP genotyping of one to several thousands of SNPs can be performed simultaneously using various chemistries such as Taqman® probes [53,54], Invader® [55], iPLEX® [56], KASPar^TM^[57], SNaPshot^TM^[58], GoldenGate® [59] and Infinium® assays [60]. The high throughput and constantly decreasing cost of sequencing technologies makes genotyping-by-sequencing (GBS) an attractive choice for genome-wide SNP genotyping.

The objective of the current study was to discover and validate SNPs in flax using a combined NGS of RRLs and GBS strategy with the updated annotation based genome-wide SNP discovery pipeline (AGSNP) [34,61]. The resource promises to have several downstream applications including the exploitation of flax genetic diversity through the understanding of important phenotypic traits.

## Results

### Selection of genotypes, sequencing and sequence alignment

Flax genotypes CDC Bethune, Macbeth, SP2047 and UGG5-5 were selected because they are parents of mapping populations. Atlas, Double Low, G-1186/94 and Crepitam Tabor were chosen from a core collection of flax lines because they were genetically diverse according to our previous assessment based on several hundred SSR markers [62]. The broader genetic diversity of these lines minimizes potential biases caused by breeding selection which could decrease the usefulness of SNPs in association mapping [63].

**Figure 1:**
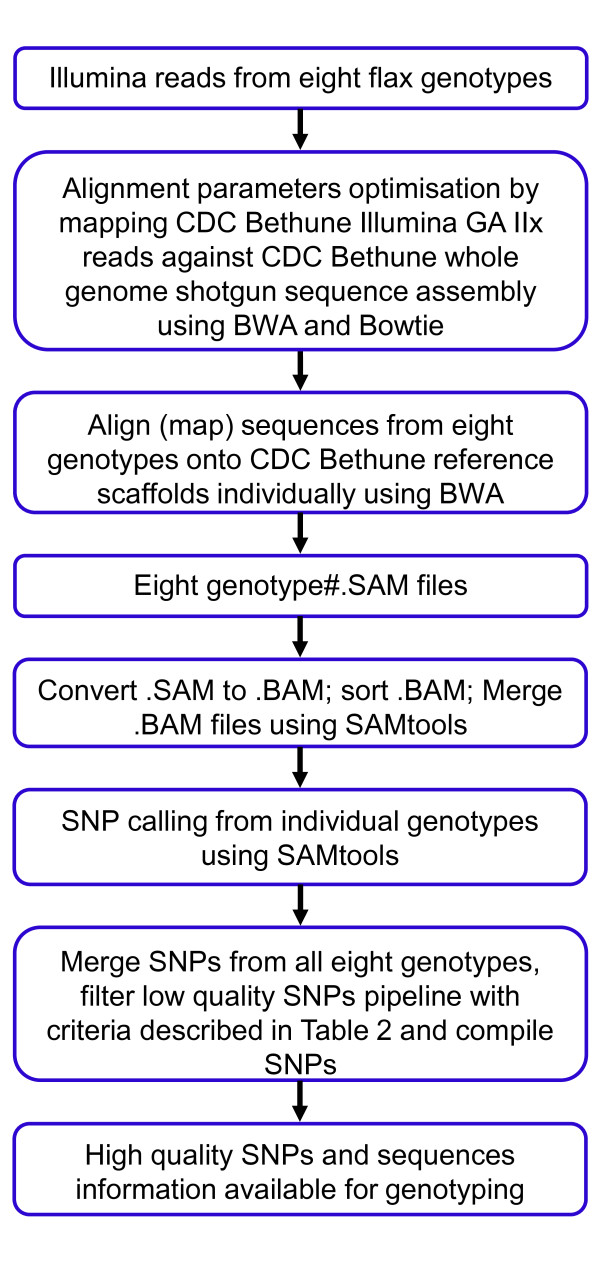
SNP discovery pipeline using Illumina GAIIx sequence reads of eight flax genotypes aligned against the whole genome shotgun sequence assembly of CDC Bethune.

All sequencing was performed on the Illumina platform. Advances in sequencing technology through the duration of the project led to reads ranging in length from 50 to 100 bp, thus resulting in variation in the number of paired end tag (PET) reads and in sequence coverage among the eight genotypes (Table 1). The data was deposited in the Sort Read Archive of NCBI under accession number SRA061924. The SNP discovery procedure is illustrated in Figure 1.

**Table 1:** Summary of sequencing and read mapping of the Illumina GAIIx reads of the reduced representation libraries of eight flax genotypes

**Genotype**	**Read length (bp)**	**Total**			**Mapped**		**Unmapped**		**Average mapped read depth**^**a**^	**Average mapping coverage (%)**^**b**^
		**Number of PET reads**	**Length (Mbp)**	**Genome equivalent (X)**	**Number of PET reads (%)**	**Length (Mbp)**	**Number of PET reads**	**Length (Mbp)**		
CDC Bethune	50	34,290,788	1,715	5	31,817,354 (93)	1,591	2,473,429 (7)	124	11.20	8.83
Macbeth	50	32,815,888	1,641	4	30,297,145 (92)	1,515	2,518,739 (8)	126	7.86	13.97
SP2047	50	35,570,612	1,779	5	32,667,382 (92)	1,633	2,903,225 (8)	145	11.41	9.29
UGG5-5	50	32,046,570	1,602	4	29,139,577 (91)	1,457	2,906,989 (9)	145	10.24	8.62
Double Low	75	56,669,792	4,250	12	47,272,267 (83)	3,545	9,397,514 (17)	705	9.28	26.65
Crepitam Tabor	75	57,974,144	4,348	12	46,740,722 (81)	3,506	11,233,410 (19)	843	7.95	37.04
G-1186/94	100	57,867,644	5,787	16	45,245,067 (78)	4,525	12,622,561 (22)	1,262	9.61	26.77
Atlas	100	56,900,660	5,690	15	45,599,045 (80)	4,560	11,301,600 (20)	1,130	9.66	31.56
Total		364,136,098	26,812	73	308,778,559	22,332	55,357,467	4,480		

Read mapping was performed against the whole genome shotgun sequence assembly (LinUsi_v1.1) of CDC Bethune using BWA.

^a^ Average mapped read depth ($\bar{X}$)was based on a fitted extreme value distribution of mapped read depth in a bin width of 1 Kbp sequence.

^b^ The average mapping coverage percentage was estimated based on the CDC Bethune reference genome sequence length of 302 Mbp (size of scaffolds without gaps).

Bowtie [64] and BWA algorithms [65] were used to map Illumina reads from the eight genotypes to the CDC Bethune whole genome shotgun (WGS) sequence assembly (LinUsi_v1.1, NCBI genome project #68161) [8], hereafter referred to as the ‘reference sequence’. For CDC Bethune Illumina PET reads, the Bowtie algorithm mapped approximately 60.4% of the reads to the reference sequence, 16.8% of the reads were supressed due to more than one reported mapping location and 22.8% of the reads remained unmapped (Additional file 1). Overall, 50.9% of the reads from the eight genotypes mapped to the reference sequence using Bowtie (Additional file 1). Using BWA, the 34.2 million CDC Bethune reads resulted in 31.8 million mapped reads (93%) with 2.5 million remaining unmapped (7%) (Table 1), thus showing the highest percentage of mapped reads as was expected because the reference sequence was obtained from this genotype. Out of 364 million combined reads from the eight genotypes, the BWA algorithm mapped approximately 309 million reads (84.8%) and 55 million reads (15%) remained unmapped (Table 1). The percentage of mapped reads ranged from ~78 % to 93% depending on the genotypes. The maximum sequence coverage was obtained from G-1186/94 followed by Atlas with 16X and 15X, respectively (Table 1).

**Figure 2:**
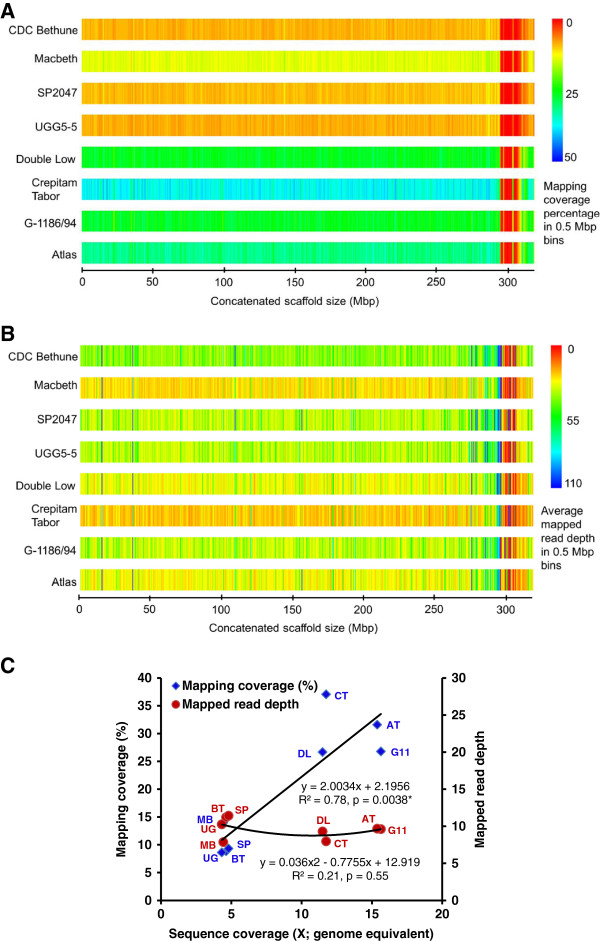
**Mapping characteristics of Illumina reads of eight flax genotypes in 0.5 Mbp bins of the concatenated CDC Bethune whole genome shotgun sequence assembly.** (**A**) Heat map distribution of mapping coverage percentage (MCP) and (**B**) average mapped read depth (MRD). The heat maps were generated using an in-house Java based program. (**C**) Relationship of sequence genome coverage (X; genome equivalent) with MCP and average MRD (BT-CDC Bethune, MB-Macbeth, SP-SP2047, UG-UGG5-5, DL-Double Low, CT-Crepitam Tabor, G11-G-1186/94, AT-Atlas).

The distribution of the mapping coverage percentage (MCP) and the mapped read depth (MRD) in bins of 0.5 Mbp over the entire length of the concatenated reference sequence is shown as heat maps in Figures 2A and 2B, respectively. PET reads from RRL sequences were distributed throughout the concatenated reference sequence, except for the tail end regions that consisted of short sequence contigs less than 200bp in length. A significant positive correlation (R^2^=0.78, P=0.0038*) between MCP and sequence coverage was observed (Figure 2C). However, the MRD remained relatively unchanged with an increase in sequence coverage, showing no significant correlation between MRD and sequence coverage (R^2^=0.21, P=0.55ns).

### SNP discovery and characterization

**Figure 3:**
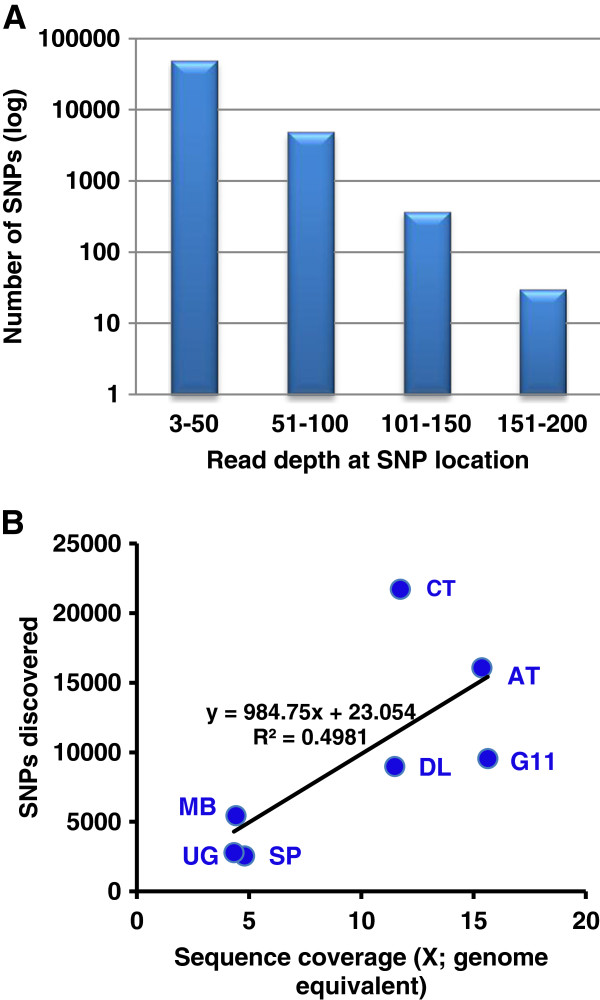
**Relationship of SNP discovery with sequence coverage and read depth in seven flax genotypes.** (**A**) Read depth frequency distribution of 55,465 SNP locations identified by alignment of Illumina GAIIx reads of seven genotypes against the CDC Bethune whole genome shotgun sequence assembly. A minimum of three reads per genotype was required for SNP calling. A log scale was used for the number of SNPs because of the disproportion in the 3-50 reads bin. (**B**) Correlation of SNP discovery with sequence coverage expressed as genome equivalents (BT-CDC Bethune, MB-Macbeth, SP-SP2047, UG-UGG5-5, DL-Double Low, CT-Crepitam Tabor, G11-G-1186/94, AT-Atlas).

The alignment file generated by BWA was used as input for SNP discovery using SAMtools [66]. A total of 71,128 putative SNPs with a quality score ≥ 20 were identified and processed through the modified AGSNP pipeline [34,61]. After applying the stringent SNP filtering criteria described in Table 2, a total of 55,465 SNPs were retained. The majority of the SNPs (~90%) from the seven contrasting genotypes were represented by read depths of ≤ 50 and 10% were discovered in regions with total read depths between 51 and 200 (Figure 3A). Sequencing and/or mapping errors (false positives) were estimated by counting the single nucleotide mismatches generated by mapping the CDC Bethune PET reads onto the reference sequence. A false positive rate of 1.9×10^-5^ per nucleotide of the reference sequence (6,072 polymorphic sites/318 Mbp) was obtained using BWA and SAMtools. 

**Table 2:** SNP filtering criteria for SNP discovery

**Criteria used for SNP calling**	**Cut-off values**
Minimum mapped read depth to the reference	≥ 3
Maximum mapped read depth to the reference	$\bar{X}$ + 2*s*^a^
Consensus base ratio	≥ 0.9
Mapping quality score in SAMtools	≥ 20
Removal of homopolymer SNPs with base string length	≥ 3 bp
Removal of very close SNPs with gap between contiguous SNPs	< 2 bp

^a^$\bar{X}$ + 2*s* is the average read depth and standard deviation estimated based on the fitted extreme value distribution for each genotype separately.

**Figure 4:**
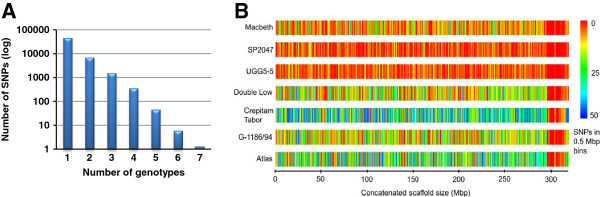
**SNP distribution across genotypes.** (**A**) Number of genotypes displaying SNPs compared to the CDC Bethune whole genome shotgun sequence assembly. (**B**) Heat map showing the distribution of SNPs of the seven flax genotypes along the 0.5 Mbp bins of concatenated whole genome shotgun sequence assembly of CDC Bethune.

The largest number of SNPs was identified from Crepitam Tabor (21,704) followed in decreasing order by Atlas, G-1186/94, Double Low, Macbeth, UGG5-5 and SP2047 (Table 3). The SNP counts and sequence coverage were significantly positively correlated (Figure 3B). Based on the gene prediction database (http://www.phytozome.net/flax) for the reference sequence, we found that a quarter of the SNPs were present in genic regions (13,367), of which 4,515 (8%) were present in the coding regions (Table 3). The average rate of SNP discovery was one SNP per 34,888 bp for genic regions, one SNP per 11,339 bp for intergenic regions and one SNP per 8,552 bp for the entire genomic regions (data not shown). Close to 84% of the SNPs (46,428) were detected in a single genotype as compared to the reference sequence with the remaining 16% (9,037) called in two to seven genotypes (Figure 4A). The distribution of SNPs in bins of 0.5 Mbp showed that the SNPs were distributed throughout the reference genome with the exception of the small contigs as was observed for MCP and MRD (Figure 4B). High SNP density across the genome and spots of very high SNP density can be visualised on the heat maps of Crepitam Tabor and Atlas which had the most SNPs.

**Table 3:** Filtered SNPs identified from eight flax genotypes and their distribution in different genomic regions

**Genotype**	**Identified SNPs**	**Inter-genic**	**Genic**	**CDS**
Macbeth	5,436	4,065	1,371	411
SP2047	2,530	1,942	588	203
UGG5-5	2,759	2,042	717	257
Double Low	8,951	6,793	2,158	739
Crepitam Tabor	21,704	16,724	4,980	1,463
G-1186/94	9,522	7,120	2,402	955
Atlas	16,055	12,037	4,018	1,553

Classification of SNPs based on base changes included 36,156 (65.2%) transitions and 19,309 (34.8%) transversions with a transition to transversion ratio of 1.87 (Additional file 2). An approximately equal number of A/G and C/T transitions were observed while G/T and A/C transversions slightly exceeded A/T and C/G transversions.

### Validation of flax SNPs

In order to validate the SNPs, we used 5,436 SNPs identified between CDC Bethune and Macbeth (Table 3), and SNP data from the GBS of the 96 F_6_-derived RILs obtained from a cross between the same two genotypes. The 100bp PET reads of the RILs were mapped to the reference sequence and SNPs called using the same pipeline and criteria (Figure 1, Table 2). A total of 4,863 SNPs out of 5,436 SNP locations were considered for validation because these locations had mapped reads from the individuals of the RIL population that met the criteria for true and false SNPs described below.

SNP locations with reads from 86 or more RILs (90% of population individuals) that did not show segregation constituted non-validated SNPs. SNP locations with reads from the RIL population that segregated for the SNPs previously identified between CDC Bethune and Macbeth constituted validated SNPs. A total of 4,706 SNPs (96.8%) were thus validated and 157 SNPs remained non-validated.

## Discussion

The current study was undertaken to discover SNPs using flax genotypes that were parents of mapping populations and/or of diverse genetic backgrounds. The Illumina platform was chosen to sequence the RRLs because of its throughput, relatively low cost, indexing and PET capabilities.

The mapping of sequence reads was performed with Bowtie and BWA using their default settings on the same Linux based servers. Wang and colleagues suggested using Bowtie or BWA for fast and efficient alignment of Illumina short reads [67]. The current study found BWA to be superior to Bowtie at mapping short reads. A higher percentage of read mapping was achieved using BWA (84.8%) compared to Bowtie (62.3% including the supressed reads) when Illumina reads of eight genotypes were mapped onto the reference sequence (Table 1 and Additional file 1). Since most of the critical parameters between Bowtie and BWA are identical or similar, we hypothesize that the read mapping differences are likely the results of the inability of Bowtie to deal with gapped alignment, a feature incorporated in BWA. The BWA based assembly was chosen for downstream analysis because it produced higher percentages of mapped reads. However, not all but approximately 93% of the CDC Bethune reads mapped to the reference sequence which is also from CDC Bethune. Inaccuracies in the reference genome assembly, sequencing errors and incomplete reference genome sequences may have contributed to the non- or mis-alignment of reads affecting the mapping percentage [68].

The AGSNP pipeline was initially designed for large-scale genome-wide SNP discovery in large and complex genomes using next generation sequences of two homozygous lines [61]. This pipeline was successfully used for SNP discovery between two inbred lines in *Aegilops tauschii* (genome size of 4.02 Gbp). Half a million SNPs with a validation rate of 85.9% were discovered [61]. In the current study, we further updated the pipeline to simultaneously process Illumina reads from eight genotypes. A total of 55,465 SNPs were discovered with sequence data corresponding to coverage of 4.3-15.6X genome equivalents. A SNP validation rate of 96.8% indicated that the AGSNP pipeline is a high-throughput SNP discovery tool that can be applied to SNP discovery in two or more genotypes from low to high complexity genomes. The updated AGSNP pipeline is available at http://avena.pw.usda.gov/wheatD/agsnp.shtml.

The RRL approach was successfully adopted in various SNP studies [22,51,69], however, there is little information available regarding the genomic distribution of mapped reads from these studies. Our study demonstrates that the sequencing of RRLs generates reads that were distributed throughout the concatenated reference assembly making these libraries suitable for ‘genome-wide’ SNP discovery and their downstream mapping applications (Figure 2). Increasing the sequence coverage (or number of reads) did not increase the mapped read depth but significantly improved the mapping coverage percentage, eventually resulting in more SNPs discovered (Figures 2 and 3). The lack of sequences pertaining to a specific part of the concatenated assembly may also be due to the exclusion of genomic regions from the restriction digest by *Mse*I. To confirm the genome-wide distribution of the SNPs discovered in our study, we estimated the SNPs distribution in 0.5 Mbp bins and found that the SNPs were well distributed throughout the concatenated reference sequence assembly (Figure 4B).

Advances in next generation sequencing are constantly reducing the cost and increasing both the length and throughput of sequencing to the point where GBS has become possible for a large number of genotypes such as core collections or segregating populations as well as complex genomes. The use of the RRL approach has enhanced read usefulness and assisted in addressing some of the computational challenges for alignment onto a reference sequence.

In our study, 3.2% (157) of the SNPs could not be validated and were considered false-positive. The false-positive SNPs from non-repetitive regions could result from gene family or duplicate genes which can cause mis-mapping of reads. Validation failure could also be due to errors of the WGS sequence assembly or sequencing errors of the Macbeth reads. By using next generation sequencing, the current study discovered a significant number of flax SNPs with a high validation rate achieved through GBS, which was revealed to be an effective method for large scale SNP validation when used in conjunction with a segregating population. The RRL coupled with GBS approach has been effective in maize, a large genome species of 2.3 Gbp, and barley where SNP validation rates of 91% and 99% were achieved, respectively [70].

The current study estimated the rate of SNP discovery in flax to be 0.17 SNPs per Kbp across the eight genotypes sequenced. This is lower than potato (11.5 per Kbp) [71], maize (8.9 per Kbp) [72], globe artichoke (5.6 per Kbp) [36], rapeseed (2.2 per Kbp) [37] and grapevine (2.5 per Kbp) [73] but is similar to that found in tomato (0.6 per Kbp) [74] and sweet pepper (1.0 per Kbp) [75]. The lower SNP rate may reflect the low sequence coverage (4-5X) in four of the eight genotypes used or the fact that fewer genotypes were used in the current study compared to other species. The SNP discovery in genic sequences was four fold less than intergenic regions possibly because the intergenic regions evolve faster and accumulate higher polymorphism compared to the conserved genic regions [76]. SNPs from the intergenic regions can, however, also be functional because some non-coding regions harbour regulatory elements like the *vegetative to generative transition 1* (*vgt1*) in maize that are crucial for flowering [77]. In addition, those intergenic SNPs are useful for the construction of high density SNP maps. The high transition/transversion ratio of 1.8 observed in the current study may be an indication of low genetic divergence [78] which can be an outcome of the self-pollinated nature of flax.

## Conclusions

Combined RRL and next generation Illumina sequencing were successfully applied for the large-scale discovery of ~55K flax SNPs that were well distributed throughout the genome. The ever decreasing cost of next generation sequencing combined with an ability to index multiple lines per lane enabled validation of a large number of SNPs (4,706) with a validation rate of 96.8% using GBS of a segregating population, proving this strategy to be powerful for validation purposes. These SNPs will be applied in genetic mapping, anchoring of genetic maps with WGS sequence assembly, marker-assisted selection, association mapping and phylogenetic analysis and, as such, they will constitute an important genomic resource for flax studies.

## Methods

### Genetic material and DNA isolation

Eight flax genotypes namely CDC Bethune, Macbeth, SP2047, UGG5-5, Atlas, Double Low, G-1186/94 and Crepitam Tabor were selected. CDC Bethune is a high yielding oilseed flax variety with intermediate oil content, oil quality, seed size and resistance to lodging, rust and fusarium wilt [79]. Macbeth is a medium to late maturing variety that is also lodging resistant and has good yield, high oil content and good oil quality. It is resistant to various forms of rust, fusarium wilt and powdery mildew [80]. SP2047 (Linola^TM^ 2047) is a yellow-seeded solin line characterized by low linolenic acid (LIN) content (2-4%) [81] whereas UGG5-5 is a brown-seeded breeding line with higher LIN content (63-66%) than conventional flax varieties such as CDC Bethune and Macbeth. Double Low is a yellow seeded oilseed breeding line which is low in the two major seed forms of cyanogenic glucosides, namely linustatin and neolinustatin. G-1186/94 is a German yellow seeded oilseed breeding line. Atlas is Swedish flax variety released more than half a century ago [82]. Crepitam Tabor is a Hungarian fibre flax genotype.

The plants were grown in pots in a greenhouse with a 16 h light and 8 h dark cycle. DNA was extracted from 10 mg of lyophilised leaf tissue using the Qiagen DNeasy 96 plant kit (Qiagen Sciences, Maryland, USA) according to manufacturer’s instructions. A total of 8 4bp cutter restriction enzymes were evaluated for their ability to restrict flax genomic DNA. The enzyme *Mse*I was selected because it yielded a large fraction of DNA smaller than the 500bp target size and it generated few high copy number bands in this region. A total of 20 μg of DNA of each genotype was restricted with *Mse*I (New England Biolabs, Beverly, MA, USA) according to the manufacturer’s instructions. The digested DNA was separated on a 1.6% agarose gel for 6 h at 100 volts and fragments in the 350-425 bp size range were excised. This size range was spanned by two high copy number bands that were not included in the excised fraction. Gel extraction of the DNA fraction was done with the QIAEX II gel extraction kit (Qiagen Sciences).

### Illumina sequencing

RRL construction from the 350-425bp fraction and Illumina/Solexa sequencing [83] was performed using Illumina GAIIx sequencing platform (Illumina Inc., San Diego, USA) by the Michael Smith Genome Sciences Centre of the BC Cancer Agency, Genome British Columbia (Vancouver, BC, Canada). Four of the libraries were sequenced as 50bp, 2 as 75bp and 2 as 100bp PET (Table 1).

### SNP discovery and characterization

The WGS sequence assembly of CDC Bethune (http://www.phytozome.net/flax, NCBI genome project #68161) [8] was used as reference for mapping of all sequence reads. Reads from all eight genotypes including CDC Bethune were aligned using Bowtie (version 0.12.8) and BWA (version 0.6.1) using default settings. An additional parameter to report only the uniquely mapped reads (m=1) was added in Bowtie. The software package SAMtools was used to convert the sequence alignment files from sequence alignment/map (SAM) to sorted binary alignment/map (BAM). The pileup files containing the SNPs were processed through an updated AGSNP pipeline [61] to filter SNPs that had a minimum of three occurrences within any accession. The method is outlined in Figure 1.

Sequence coverage expressed as genome equivalents (X) was calculated by dividing the total read length by the estimated size of the flax genome (~370 Mbp) [43,44]. Mapping coverage percentage (MCP) and mapped read depth (MRD) were used to characterize the sequence coverage and average read mapping depth within a defined interval of the concatenated reference sequence referred to as bins. MCP represents the percentage of the reference sequence (318 Mbp) covered by reads of individual genotypes within a bin size of 0.5 Mbp. Similarly, MRD is the average number of mapped reads per mapped position within a bin size of 0.5 Mbp from the individual genotypes mapped separately onto the reference sequence. The heat maps showing MCP, MRD and SNP density were generated using an in-house program written in Java.

In the original AGSNP pipeline [61], the average mapped read depth ($\bar{X}$) was used to identify single copy reference sequences and to set a maximum read depth for filtering paralogous genes or repetitive sequences. Average mapping read depth plus 2 times its standard deviation (s), $\bar{X}$ + 2*s*, was considered to be an optimal cut-off value in the self-pollinating species *Aegilops tauschii*. We used the same criteria for flax, applying it to each genotype to remove potentially false-positive SNPs due to highly repetitive sequences or mis-mapping. $\bar{X}$ and *s* were estimated for each genotype based on the mapping results using a pipeline program in the AGSNP package. The SNP filtering criteria are listed in Table 2.

To determine SNP location within genes, we used the gene prediction database available at http://www.phytozome.net/flax that was created using Augustus (version 2.5.5), a Hidden Markov Model-based gene finding program [84] and Glimmer HMM (version 3.0.1) [85].

### SNP validation

GBS was performed on a 96 F_6_-derived CDC Bethune/Macbeth RIL population. RRLs were constructed for each RIL and four RILs were indexed per lane of Illumina GAIIx. The 100bp PET reads were mapped onto the CDC Bethune reference sequence assembly using the modified AGSNP pipeline and the same parameters as described earlier for the eight genotypes (Table 2). The SNP list generated from the 96 RILs was used to confirm the SNPs initially discovered with the parental accessions. The validation strategy is detailed in the results section of this manuscript.

## Competing interests

The authors declare that they have no competing interests.

## Authors’ contributions

SK participated in the design of the study, carried out bioinformatics analysis and wrote the manuscript. FY participated in bioinformatics analysis. SC designed and supervised the study and co-wrote the manuscript. All authors read and approved the final manuscript.

## Supplementary Material

Additional file 1 **Read lengths and mapping results from the Illumina GAIIx reads of the reduced representation libraries of eight flax genotypes.** Read mapping was performed against the new whole genome shotgun sequence assembly (LinUsi_v1.1) of CDC Bethune using Bowtie. Click here for file

Additional file 2 Transition and transversion frequencies of the 55,465 SNPs. Click here for file
