# Supplementary material for: Genome wide SNP discovery in flax through next generation sequencing of reduced representation libraries
Source: BMC Genomics. 2012 Dec 6;13:684. doi: 10.1186/1471-2164-13-684 (PMC3557168; doi:10.1186/1471-2164-13-684)
Supplement: Additional file 1 — Read lengths and mapping results from the Illumina GAIIx reads of the reduced representation libraries of eight flax genotypes. Read mapping was performed against the new whole genome shotgun sequence assembly (LinUsi_v1.1) of CDC Bethune using Bowtie. [file 1471-2164-13-684-S1.pdf]

Additional File 1 - Read lengths and mapping results from the Illumina GAIIx reads of the reduced representation libraries of eight flax genotypes. Read mapping was performed against the new whole genome shotgun sequence assembly (LinUsi_v1.1) of CDC Bethune using Bowtie.

Genotype	Read length (bp)	Mapped		Unmapped		Suppressed		Total	
		Number of PET reads (percent)	Length (Mbp)	Number of PET reads (percent)	Length (Mbp)	Number of PET reads (percent)	Length (Mbp)	Number of PET reads (pairs)	Length (Mbp)
CDC Bethune	50	10,353,767(60.4)	1,035	3,905,439 (22.8)	391	2,886,188 (16.8)	289	17,145,394	1,715
MacBeth	50	9,884,782 (60.2)	988	4,152,965 (25.3)	415	2,370,197 (14.4)	237	16,407,944	1,641
SP2047	50	9,535,745 (53.6)	954	5,091,315 (28.6)	509	3,158,246 (17.8)	316	17,785,306	1,779
UGG5-5	50	9,104,662 (56.8)	910	4,501,814 (28.1)	450	2,416,809 (15.1)	242	16,023,285	1,602
Double Low	75	14,214,503 (50.2)	2,132	11,444,119 (40.4)	1,717	2,676,274 (9.4)	401	28,334,896	4,250
Crepitam Tabor	75	14,615,616 (50.4)	2,192	11,957,292 (41.3)	1,794	2,414,164 (8.3)	362	28,987,072	4,348
G-1186/94	100	12,686,427 (43.8)	2,537	13,460,180 (46.5)	2,692	2,787,215 (9.6)	557	28,933,822	5,787
Atlas	100	12,405,940 (43.6)	2,481	13,987,831 (49.2)	2,798	2,056,559 (7.2)	411	28,450,330	5,690
Total		92,801,442	13,229	68,500,955	10,766	20,765,652	2,815	182,068,049	26,812
