# Supplementary figures and images for: Genome wide SNP discovery in flax through next generation sequencing of reduced representation libraries
Source: BMC Genomics. 2012 Dec 6;13:684. doi: 10.1186/1471-2164-13-684 (PMC3557168; doi:10.1186/1471-2164-13-684)

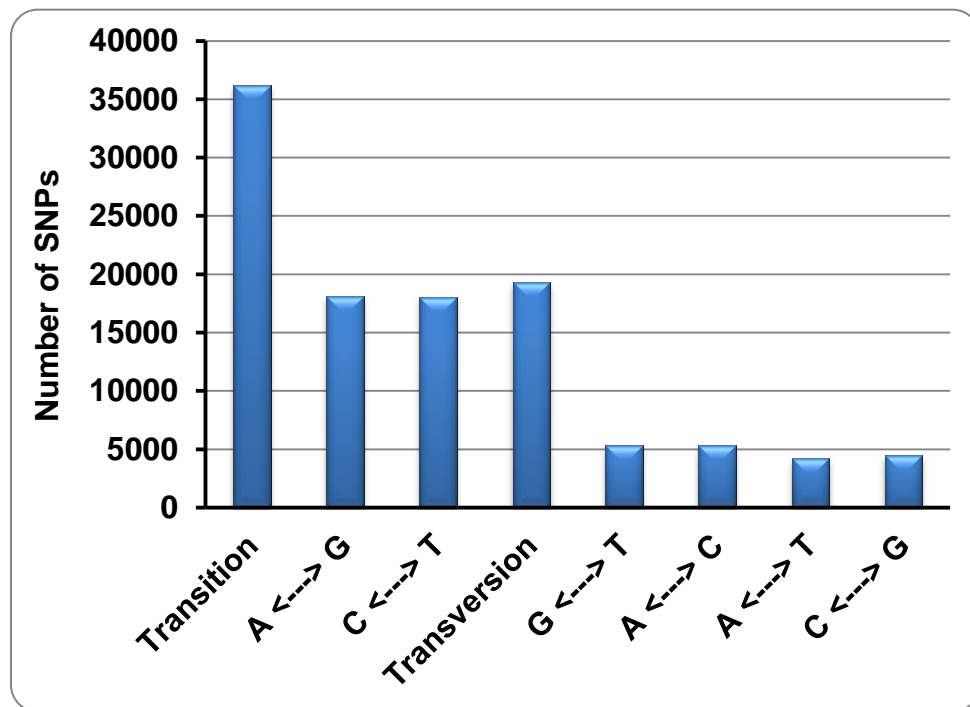

**Additional File 2** - Transition and transversion frequencies of the 55,465 SNPs.

Supplement: Additional file 2 — Transition and transversion frequencies of the 55,465 SNPs. [file 1471-2164-13-684-S2.pdf]
